# COMPARABILITY OF BIOLOGICAL AGING MEASURES IN THE NATIONAL HEALTH AND NUTRITION EXAMINATION STUDY, 1999-2002

**DOI:** 10.1093/geroni/igz038.1783

**Published:** 2019-11-08

**Authors:** Waylon J Hastings, Daniel Belsky, Idan Shalev

**Affiliations:** 1 Pennsylvania State University, State College, Pennsylvania, United States; 2 Columbia University Mailman School of Public Health, New York, New York, United States

## Abstract

Biological processes of aging are thought to be modifiable causes of many chronic diseases. Measures of biological aging could provide sensitive endpoints for studies of risk factors hypothesized to shorten healthy lifespan and/or interventions that extend it. However, uncertainty remains about how to measure biological aging and if proposed measures assess the same thing. We tested four proposed measures of biological aging with available data from NHANES 1999-2002: Klemera-Doubal method (KDM) Biological Age, homeostatic dysregulation, Levine Method (LM) Biological Age, and leukocyte telomere length. All measures of biological aging were correlated with chronological age. KDM Biological Age, homeostatic dysregulation, and LM Biological Age were all significantly associated with each other, but were each not associated with telomere length. NHANES participants with older biological ages performed worse on tests of physical, cognitive, perceptual, and subjective functions known to decline with advancing chronological age and thought to mediate age-related disability. Further, NHANES participants with higher levels of exposure to life-course risk factors were measured as having older biological ages. In both sets of analyses, effect-sizes tended to be larger for KDM Biological Age, homeostatic dysregulation, and LM Biological Age as compared to telomere length. Composite measures combining cellular- and patient-level information tended to have the largest effect-sizes. The cellular-level aging biomarker telomere length may measure different aspects of the aging process relative to the patient-level physiological measures. Studies aiming to test if risk factors accelerate aging or if interventions may slow aging should not treat proposed measures of biological aging as interchangeable.

